# A sonic root detector for revealing tree coarse root distribution

**DOI:** 10.1038/s41598-020-65047-4

**Published:** 2020-05-15

**Authors:** Andrea R. Proto, Antonino Di Iorio, Lorenzo M. Abenavoli, Agostino Sorgonà

**Affiliations:** 1 Department of Agriculture, Mediterranean University of Reggio Calabria, Feo di Vito, 89122 Reggio Calabria, Italy; 2 Department of Biotechnologies and Life Sciences, University of Insubria, 21100 Varese, Italy

**Keywords:** Plant sciences, Environmental impact

## Abstract

This study assesses the reliability of a non-destructive method for determining the *in situ* distribution of tree coarse roots within a scaled distance 6-fold the DBH by comparing the results with the actual 3D root architecture revealed by invasive methods. The root architecture of 22-year-old olive trees was determined non-destructively with a Root Detector device (Fakopp Enterprise Bt) using sonic speed and directly by a 3D digitizer (Fastrak, Polhemus) after soil removal. The radial and vertical distributions of the coarse root biomass and diameter in the soil as determined by the 3D digitizer were correlated with the root map detected by sonic speed. A highly significant correlation was observed between the coarse root biomass distribution and the sonic speed within 30 cm of soil depth, but this correlation decreased with increasing distance from the trunk, up to 120 cm. No correlations were observed between sonic speed and root diameter. The Root Detector was able to map the coarse roots of the olive tree in the soil environment, but only under certain conditions. First, root detection was more efficient within 30 cm of soil depth, provided that more than 35% of the total biomass of lateral roots occurs within this depth range. Second, the distance of 120 cm from the trunk, scaled as 6-fold the DBH, may be considered the threshold over which the sonic speed and the detection of roots markedly decreased. Third, Root Detector technology is unable to detect root size in terms of geometric parameters such as root diameter.

## Introduction

Root architecture, i.e., the root radial and vertical distribution in the soil, is involved in different plant functional roles, such as nutrient and water acquisition^1,2^ and mechanically active anchorage in the soil, which determines tree stability^3^. The importance of the mechanical role of root architecture in tree stability has been extensively described in previous studies^4–7^. The failure of urban and forest trees is often a consequence of the low performance of this root feature caused by root decay, injury, breaking or low biomechanical acclimation to, for example, wind loads^8,9^.

High frequencies of storms, windstorms and other similar extreme events are generally responsible for more than 50% of the damage by volume to European forests^13,14^ and are expected to increase under the observed ongoing climate changes^10–12^. Furthermore, tree instability threatens property and people in urban forests^15^. Hence, evaluations of root health/decay/injury and the root radial and vertical distribution in the soil in particular are crucial for both tree stability and consequently for safety in urban environments^16^.

Because roots grow belowground, the radial and vertical root distributions of trees and crop fruits are mainly determined by destructive methods that involve excavating the root system from the soil and digitizing the system using different 3D digitizers, such as magnetic (Fastrak, Polhemus, Colchester, VT, USA)^17,18^ or sonic (GTCO Freepoint 3D) 3D digitizers^19^. Following digitization, the root architecture can be reconstructed with different specific softwares: Floradig (CSIRO, Australia) or AMAPmod (AMAP, France). Fastrak-AMAPmod technology has already been used to digitize and analyse the 3D root architecture mainly of forest species (*Pinus*: 19; *Picea*: 8; *Quercus*: 18^20^,), shrubs (*Jathropa curcas*: ^21^ *Spartium junceum*: ^22^), herbs (*Zea mays*: ^23^) and fruit species (*Olea*: ^24^). Using Fastrak technology, it is possible to estimate several root biometric and geometric parameters at different soil depths and radial distributions, such as biomass, length, diameter, and angle. However, some limitations have been noted for these root techniques, such as high sensitivity to wind fluctuations in the field for the sonic digitizer^25^ and the interference with the Fastrak magnetic field by surrounding iron frames^26^. Above all, root excavation methods are extremely time consuming and labour intensive, and they are not always an option, especially in urban environments, because of the presence of pavement as well as underground service lines, pipes, rocks, and other buried materials.

For these reasons, root scientists have long sought reliable, non-destructive methods for detecting, measuring, and visualizing the hidden half of living trees, both in pot and field-grown conditions. For example, X-ray computed tomography (X-ray CT) (^27,28^ in pots; ^29^ in the field), electrical impedance tomography (EIT) (^30^ in pots; ^31^ in the field), magnetic resonance imaging (MRI) (^32^ in pots), magnetic resonance imaging-positron emission tomography (MRI-PET) (^33^ in pots) and ground-penetrating radar (GPR) (^34^ in the field) have been used for root architecture analysis. However, for CT technology, the high soil water content causes an attenuation problem, i.e., the technology can’t distinguish the root from other nonspecific soil organic matter^35^. MRI is limited by high water content, while MRI-PET is a technique under development^36^. GPR is more limited in urban environments because it fails to distinguish roots from utility lines (gas and water pipes)^35^ and is not effective in soils with high clay or water content^37^. In addition to these limitations, these techniques are also very expensive; therefore, no efficient and inexpensive non-destructive method exists for mapping tree root systems or for identifying the presence of individual large roots^38^.

Recently, based on sonic tree tomography and the principle that sound waves travel more slowly through soil than through healthy wood, two studies examined the application of this principle for the detection of coarse roots in the soil^39,40^. However, none of them provided a robust statistical investigation of the relationship between the sonic speed and the actual 3D root architecture or size-dependent morphological traits, such as biomass/volume/diameter; these studies only provided a visual assessment of the congruity between the photographed or drawn root map with the speed spectra measured at different distances and directions from the trunk base. Given these conditions, this paper details a pilot study investigating the reliability of a non-destructive method, i.e., Root Detector + ArborSonic 3D (Fakopp Enterprise Bt, Hungary), for determining the presence and radial distribution of the individual roots by statistical correlation of the sonic speed data with those obtained from the detailed root radial and vertical distribution by a 3D digitizer (3 Space Fastrak, Polhemus Inc., Colchester, VT). The maximum radial distance investigated was approximately 6-8-fold the DBH; more than 80% of the root system biomass of temperate tree species is confined within this distance (^17,18,41^ and references therein). To this aim, this paper addresses the following questions:

1. At what distance from the trunk and up to what soil depth does the Root Detector effectively detect roots?
2. What is the minimum root diameter effectively detected by the Root Detector? Of the mean diameter and the biomass of a root axis, which of the two best correlates with the Root Detector outputs?
3. Is the Root Detector able to determine the root radial distribution?

As a case study, considering that the root architecture of olive trees changes in response to different harvesting methods^24^, two individuals with hypothetically different root architecture were selected.

## Materials and Methods

### Site description and plant material

The experiments were conducted in an organic olive tree orchard in Satriano, Calabria (Italy). The general characteristics of this area were already described in a previous work carried out on the same site^24^. Briefly, the site is located on a southwest-facing slope (300 m altitude, 38°39’ N, 16°28’E, mean slope of 20°) on a clay loam, medium calcareous soil, with slightly alkaline pH. Two adult (22-year-old) olive trees *(Olea europaea* L.), hereafter named Tree09 and Tree11, were selected from an orchard planted at 6 ×5 m spacing, equal to 334 tree ha^−1^ density. The trees were obtained by grafting the Carolea cultivar onto seedlings and were trained according to the vase system^24^. The two olive trees were selected for their different harvesting methods, manual and mechanical, for Tree09 and Tree11, respectively; the olive harvesting method is known to change the root architecture^24^ (see Table 1 for aboveground tree parameters).

**Table 1:** Aboveground parameters of the two olive trees characterized by manual (Ma) and mechanical harvesting method (Me).

	DBH (at 1 m height) (cm)	Tree height (m)	Trunk height (m)	Canopy (m^2^)
Tree09 (Ma)	22	4.1	0.95	10
Tree11 (Me)	18	3.4	1.04	6

The olive trees were not irrigated. For the other crop management practices (fertilization, tillage and crop protection), both trees were treated equally following the recommended standards for organic cultivation as indicated by EU (Reg. CE 834/07 and 889/08) and national regulations (MD 18354/2009)^24^.

### Root detector

The root radial distribution was estimated *in situ* by using the Root Detector together with ArborSonic 3D, both distributed by Fakopp Enterprises Bt (Hungary). Briefly, the device consists of a control unit (ArborSonic 3D), a SD02 piezo sensor (the transmitter) with the spike inserted at the tree collar (the tapping point) and pointed towards the roots, and a soil sensor (the receiver; a high-frequency geophone) with the spike inserted into the soil (Fig. S1; the persons depicted in the photographs gave their informed consent for the publication of identifying images in an online open-access publication). The method is based on sonic tree tomography, which determines the quality of the mechanical connection between sending and receiving sensors by measuring the time-of-flight of stress waves^42,43^. This principle is also applied to root detection and is based on the acoustic signal’s velocity difference between the root and the soil: the travel time decreases significantly when the root is detected and is nearby (closer than 10 cm)^40^.

In our experiment, the measurements were performed along three circles around the olive trunk at distances of 40, 80 and 120 cm from the trunk surface (marked using lines on the ground) and at every 10 degrees, called sectors, in the clockwise direction for each circle. A further set of measurements was carried out at 160 cm, but the halved sonic speed and the increased coefficient of variation made these measurements unsuitable for the calculations. Therefore, thirty-six values for each circle for a total of 108 values (36 measurements x 3 distances) for each plant were obtained. To maintain a constant distance between the tree and the soil sensor, a loose rope was tied around the tree (Fig. S1). At each distance, and starting at the “0 degree” point, the soil sensor was moved along the circle in each sector by the “Step” value shown by the software, making sure that the rope remained taut. Three hits are necessary to obtain valid data that represent the sonic speed (m s^–1^). The estimated time per person required for setting up and recording 108 measurements was 45 minutes.

In Fig. S1, a typical output graph from the Root Detector Evaluation Software (Fakopp Enterprise Bt, Hungary) showing the data from each single measurement point coloured in grey levels is provided; the darker the grey colour, the higher the sonic speed and, consequently, the shallower and larger the root. According to the device settings, the speed values are considered relevant when they are higher than the mean value of the measured range [(min+max)/2]. However, in this study, representation with vectors, which better indicate the magnitude and direction of the sonic speed data for each 10-degree sector, was preferred.

### Excavation and 3D root architecture measurement

After measurements with the Root Detector, the same root systems were freed from soil using high-pressure air lances (AirSpade 2000, Chicopee, MA, USA) as already described in Sorgonà et al. (24 and references therein). An Air-Spade supplied with compressed air from a standard 4.25 m^−3^ min^−1^ road-works compressor loosened and removed the soil from around the trunks with little or no damage to the roots. The root systems were exposed to depths of 1 m and to distances of ≈ 1.5 m from the trunk (i.e., the bark surface) (Fig. S2; the persons depicted in the photographs gave their informed consent for the publication of identifying images in an online open-access publication). The volume of soil removed (≈ 4 m^3^) was not as large as to compromise the stability of the tree or the operator’s safety. No rotten roots were observed.

Three-dimensional position coordinates (*X, Y, Z*) of the roots with a proximal (i.e., at the branching point) diameter larger than 5 mm were measured *in situ* with a 3D digitizer (3 Space Fastrak, Polhemus Inc., Colchester, VT), a low-frequency electromagnetic field sensing-based technology (Fig. S2). The characteristics of this device are explained in previous works^17,18^. Briefly, the root system architecture was digitized by starting from the top of the stump and following a recursive path along the branching network up to a terminal diameter of 2 mm. After the 3D root architecture measurement, the root systems were covered again with the original soil to reduce disturbance.

### Root architecture analysis

**Figure 1:**
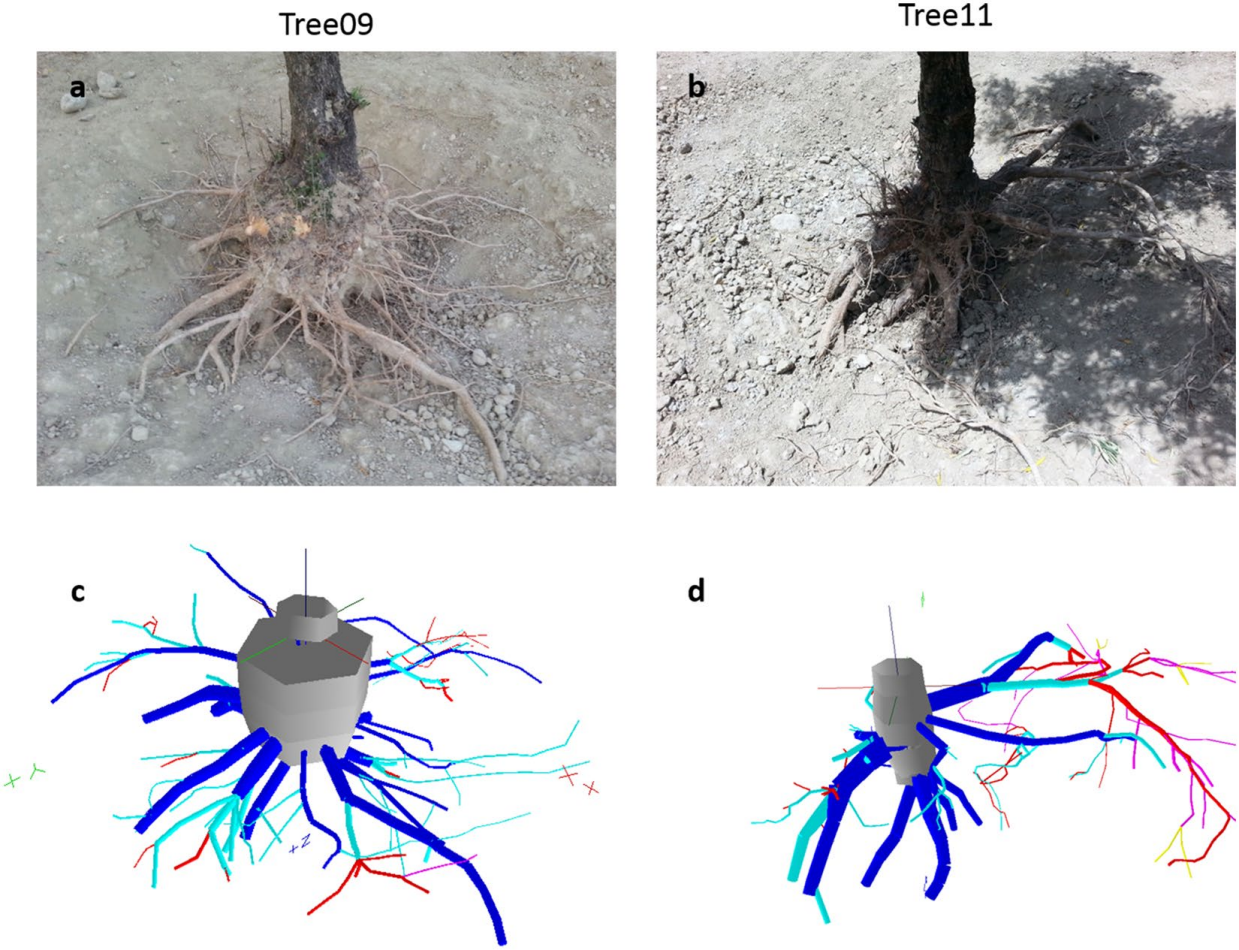
3D image reconstruction of the two studied olive root systems. Comparison between the images obtained **(a,b**) by camera and (**c,d**) by 3D reconstruction from digitization using AMAPmod software. The root axes are coloured as a function of their branching order: dark-grey, taproot; blue, first-order; light-blue, second-order; red, third-order; violet, fourth-order; yellow, fifth-order. The 3D reconstructed root systems were oriented to fit the view shown in the camera images.

The roots were assumed to be either circular or elliptical in cross-section. The geometric mean of the maximum and minimum diameters determined the mean diameter of the oval-shaped roots^24^. The output data file was analysed using AMAPmod software^25^, which handles topological structure and provides 3D graphical reconstruction for data validation (Fig. 1). All topological and geometrical data were exported to Microsoft Excel 2010 (Microsoft Office, USA) for specialized processing^22,24^. In particular, the distance between two corresponding measurement points was used as the segment length. The segment volume was calculated as a truncated cone based on its length and proximal and distal diameters. The length or volume of a root axis was the sum of the length or volume of all its segments. These computations were made with a macro suitably realized with Microsoft Visual Basic 6.0^24^.

In this work, the depth-related root detection instrument accuracy was evaluated by correlating the sonic speed data with the detailed root radial and vertical distribution obtained by the 3D digitizer. The calculations were performed using a method broadly explained in Sorgonà et al.^24^, but the specific requirements for its application in this work are provided below. Briefly, the digitized root system was virtually located in a grid made of three 30 cm thick stump centred cylinders (named D30, D60 and D90) stacked to 90 cm depth to equal the removed soil volume of ≈ 4 m^3^ (Fig. S3). The radius of the cylinders was set at the same distance as the Root Detector measurements, i.e., 40, 80 and 120 cm. The cylinders were divided into 36 sectors, 10° wide (as in the Root Detector measurements) (Fig. S3), resulting in 324 boxes (36 sectors × 3 depths × 3 distances) in total. Within each box, all the roots were virtually sliced, and their volume was determined. The root system was aligned so that the 0–30° and 330–360° sectors coincided with the downslope direction and the 150–180° and 180–210° sectors coincided with the upslope direction. The alignment matched that used for the Root Detector measurements. The root biomass was estimated by conversion using the density value empirically obtained by oven-dried root core samples extracted from roots of different diameters with an increment corer 0.5 cm in diameter (Haglöf Sweden AB, Sweden, www.haglofsweden.com). Afterwards, considering the soil volume of each sector and the soil depth, the root biomass density was calculated (root dry mass/soil volume, kg cm^−3^) at each soil depth and distance from the trunk base.

### Statistical analysis and calculation

Within each tree, the clustering tendency of the radial distribution of both the root biomass and the sonic speed values was evaluated using circular statistical methods, namely, Hotelling’s test^24,44,45^, in Oriana software v. 4.02 (Kovach Computing Services; ^46^). Hotelling’s test calculates whether the centroid of the endpoints of the weighted vectors differs from the origin, i.e., whether the weighted angles have a significant mean direction at the probability level of 0.05^24^.

To verify the detection resolution of the Root Detector, we proceeded as follows:

1. Correlation analysis (Pearson test) between the sonic speed and the root biomass density data calculated for all 36 sectors and at each distance from the trunk base and soil depths. This procedure allowed us to infer the maximum soil depth and horizontal distance from the trunk base within which the root signal is detected;
2. Correlation analysis (Pearson test) between the sonic speed and the root average diameter calculated for all 36 sectors and at each distance from the trunk base and soil depths. This analysis provided information about the efficient detection of root diameter by the Root Detector.

Considering that sound propagates in soil with three compressional waves and one shear wave (i.e., an S wave)^47,48^, and since the soil sensor of the Root Detector was inserted into the soil surface (~10 cm), the root biomass density (kg m^−3^) and the root diameter data were determined cumulatively as the sum of the volume values and the mean of the diameter values, respectively, of all the root segments located in the soil volume considered up to the selected depth and distance. For example, for the soil depth D60 and the distance from the trunk base 80 cm, all the root segments located in a soil sector 10° wide, 60 cm deep and 80 cm long were considered (Fig. S3).

Statistical analysis was conducted using SPSS Inc., V. 20.0, 2002 (SPSS Inc., Evanston, IL, USA) except where otherwise indicated.

## Results

### Root mapping by Root Detector

**Figure 2:**
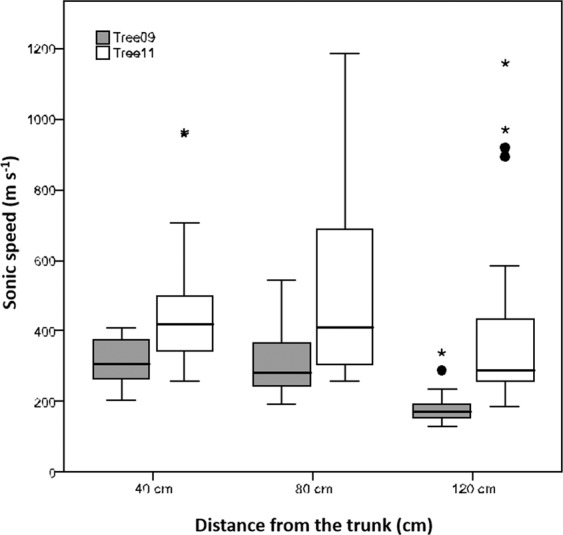
Boxplot of distributions of the sonic speed (m s−^1^) detected by Root Detector for the two *Olea europaea* trees at different distances from the trunk (0–40 cm, 0–80 cm; 0–120 cm). Circles indicate outlier values; asterisks indicate extreme values.

The Root Detector revealed sonic speeds with different magnitudes and radial distributions in both trees. For the magnitude, in particular, the lowest sonic speeds measured by Root Detector were 136 and 166 m s^−1^ for Tree09 and Tree11, respectively. A similar pattern was observed for the highest values: 581 and 1266 m s^−1^ for Tree09 and Tree11, respectively (Fig. 2). Furthermore, the sonic speed reduced its magnitude with increasing distance from the trunk, especially for Tree09, and its mean value was always significantly higher for Tree11 than for Tree09 (P < 0.05, Fig. 2), suggesting that the root system of Tree11 was shallower than that of Tree09.

**Figure 3:**
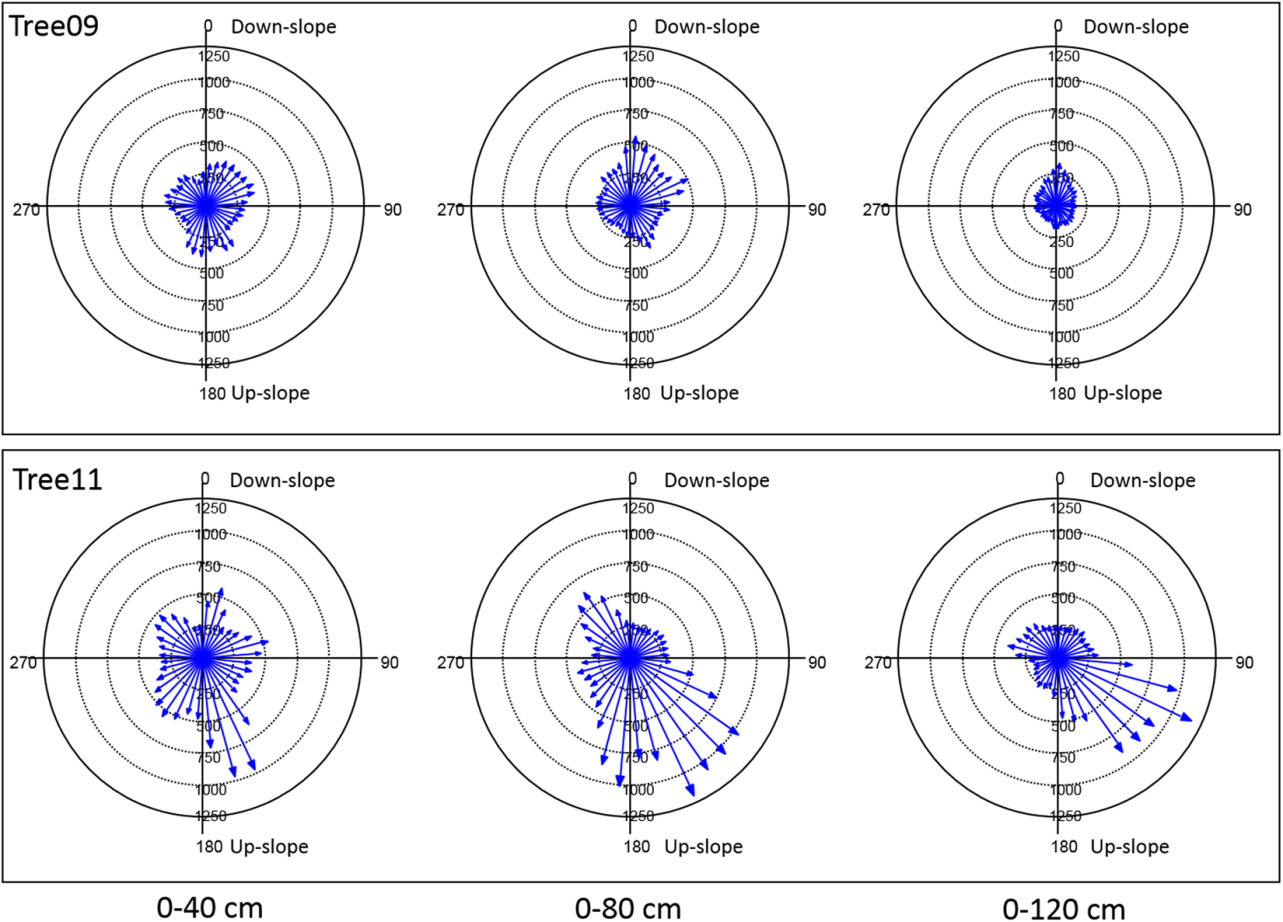
Sonic speed (m s−^1^) measured by Root Detector at different distances from the trunk (40, 80 and 120 cm) in 36 10-degree sectors for the two studied *Olea europaea* trees. The magnitude and direction of the sonic speed are represented by vectors. 0° coincides with the down-slope direction.

For the radial distribution of the roots, the Root Detector Evaluation Software provided a graphical output, as shown for Tree09 and Tree11 in Fig. S4, where circles represent the differently spaced (40, 80 and 120 cm) measurement points around the trunk, and grey levels increase with increasing sonic speed. In this work, vectors were preferred over grey levels because they better indicate the magnitude and direction of the sonic speed; these values suggest the presence of roots as well as providing a “gross” evaluation of root size (larger or smaller). For Tree09, higher sonic speed values were detected in the sectors 20°–80°/140°–210°, 0°–20°/60°–80°/150°–160°/350–360° and 0°–30°/330°–360° at 40, 80 and 120 cm of distance from the trunk, respectively (Fig. 3). The sonic speed showed a different radial distribution pattern for Tree11, specifically, sectors 10°–20°/70°–80°/140°–180°/210°–230°, 110°–200°/310°–330° and 100–160° at 40, 80 and 120 cm of distance from the trunk, respectively (Fig. 3). No preferential direction of sound propagation emerged for either tree (Hotelling’s test P > 0.05).

### 3D root architecture by Fastrak

**Figure 4:**
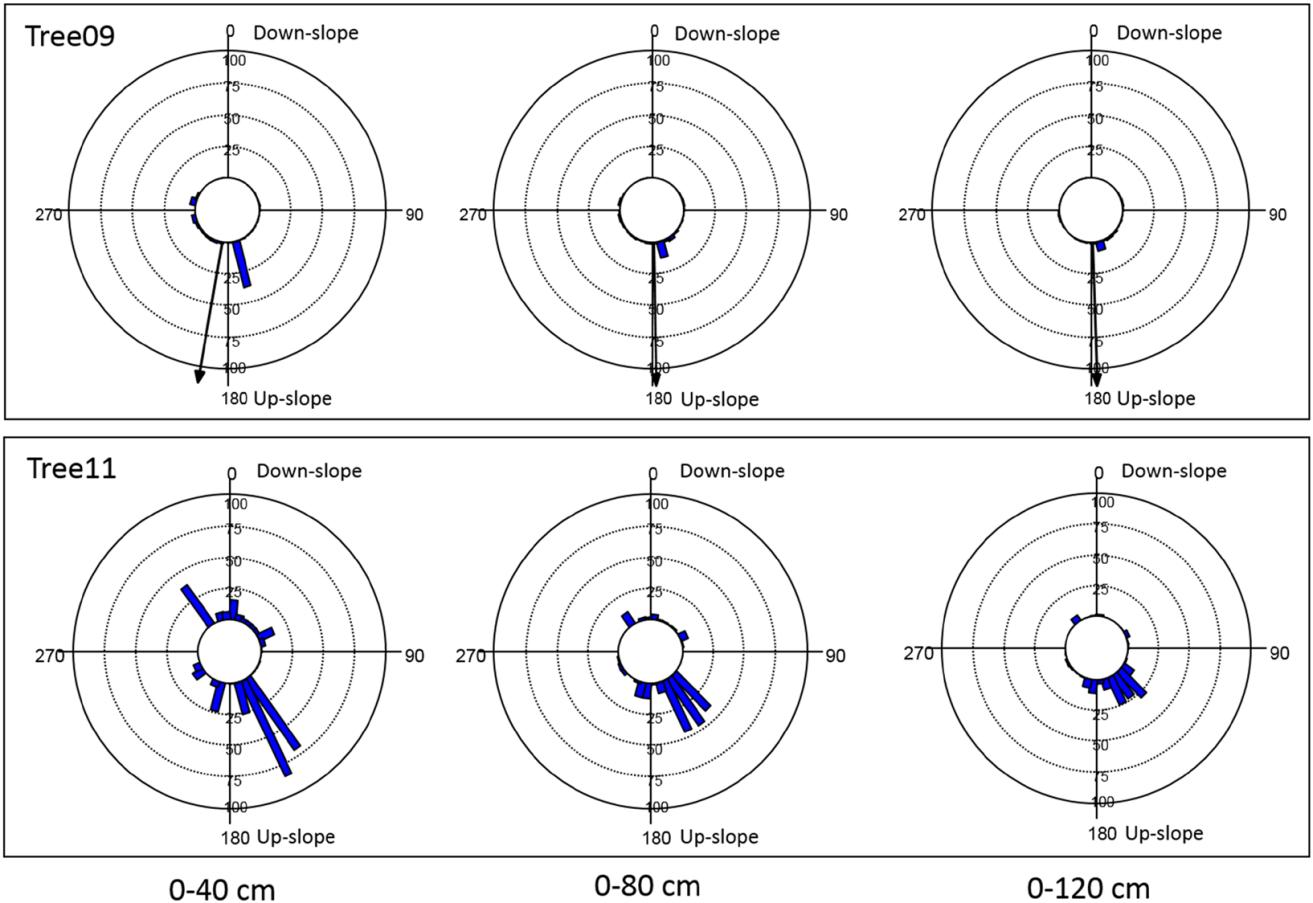
Root biomass density (kg m^−3^) distribution at different distances from the bark surface (0–40 cm, 0–80 cm; 0–120 cm) and to 30 cm soil depth within 36 10-degree sectors for the two studied *Olea europaea* trees. 0° coincides with the down-slope direction. Each bar indicates the root biomass density of the first three orders of lateral roots within the 10-degree sector. If present, the black arrow from the centre indicates the significant direction of root clustering (centre of mass).

**Figure 5:**
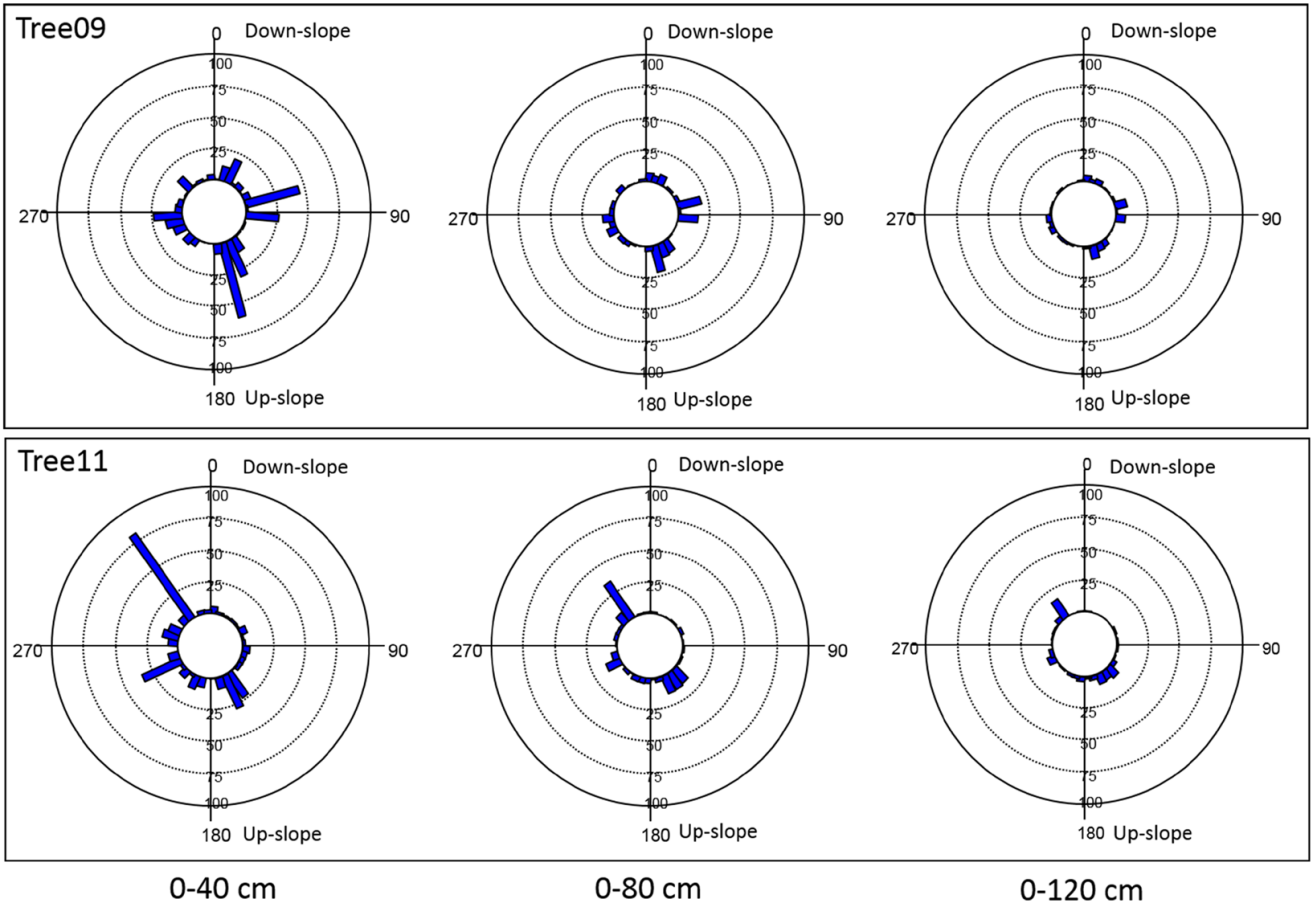
Root biomass density (kg m^−3^) distribution at different distances from the bark surface (0–40 cm, 0–80 cm; 0–120 cm) and to 90 cm soil depth within 36 10-degree sectors  for the two studied *Olea europaea* trees. 0°coincides with the down-slope direction. Each bar indicates the root biomass density of the first three orders of lateral roots within the 10-degree sector. If present, the black arrow from the centre indicates the significant direction of root clustering (centre of mass).

In this experiment, the radial distribution of the root biomass density of the two olive trees at D30 and D90 cm soil depths indicated well-defined but different root distribution patterns (Figs. 4 and 5). In particular, with 0° indicating the down-slope direction, within the shallower soil layer (D30), the root biomass significantly clustered in the 175–185° sectors for Tree09 (Hotelling’s test P < 0.05) and in the 140°–160° sectors for Tree11, although not significantly (Hotelling’s test P > 0.1) (Fig. 4). It is worth noting that neither Tree09 nor Tree11 changed their radial distribution pattern with increasing distance from the trunk (Fig. 4). Considering the whole 90 cm soil depth (D90), the root biomass of Tree09 was mainly concentrated in the 170° sector and, to a lesser extent, the 80° and 100° sectors within 40 cm from the trunk; at 120 cm distance, the 10° sector had a notable amount of root biomass (Fig. 5). Tree11 allocated its root biomass prevalently to the 330° sector, reorienting it to the 140°–160° sectors at further distances from the trunk (Fig. 5). No preferential direction of biomass allocation was observed for either tree (Hotelling’s test P > 0.05).

**Figure 6:**
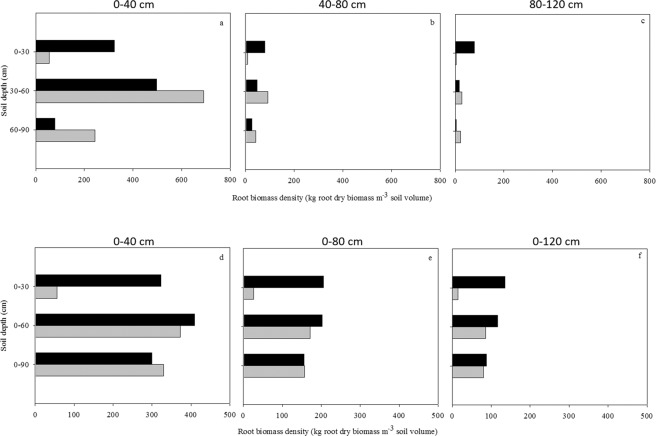
Root biomass density (kg root dry mass m^−3^ soil volume) (**a–c**) and its cumulative patterns (**d–f**) in *Olea europaea* trees (Tree09: grey; Tree11: black) with increasing soil depth [(**a**) 0–30 cm, (**b**) 30–60 cm, (**c**) 60–90 cm, (**d**) 0–30 cm, (**e**) 0–60 cm, **(f)** 0–90 cm] and distance from the bark surface [(**a**) 0–40 cm, (**b**) 40–80 cm, (**c**) 80–120 cm, (**d**) 0–40 cm, (**e**) 0–80 cm, (**f**) 0–120 cm].

In Fig. 6, the compartmentalized (Fig. 6a–c) and cumulative (Fig. 6d–f) root biomass density at different soil depths and distances from the trunk surface of the two olive trees are shown. Tree09 allocated more root biomass to the deeper soil layers preferentially at D60 and, to a lesser extent, at D90 (Fig. 6). This pattern was maintained along the distance from the trunk. Conversely, Tree11 had a preferential distribution of root biomass density in the shallower soil layers (D30) at all distances from the trunk (Fig. 6). From panels Fig. 6a–c, it is possible to infer that more than 80% of the total root biomass was located within 40 cm from the trunk base and within 0.9 m soil depth.

### Root architecture - Root Detector output data correlation analysis

Tree11 showed significant (0.001 < *P* < 0.003) coefficients of correlation (r) between the sonic speed and the root biomass density. The correlation coefficients decreased with increasing soil depth and distance from the trunk, and their highest value was calculated at 80 cm (Table 2). Conversely, Tree09 showed no correlation at any distance or soil depth. Indeed, as observed in this study, the clear detection of the root axes for Tree11 was possible because it had shallower root architecture than Tree09, which, in contrast, deepened its root axes into the soil (Fig. 6).

**Table 2:** Correlation coefficient (r) and *P* values of the correlation analysis (Pearson test) between the sonic speed and root biomass density revealed by Root Detector and Fastrak, respectively, in two olive trees at different distance from trunk (0–40, 0–80 and 0–120 cm) and soil depth (D30, D60, D90).

		0–40 cm	0–80 cm	0–120 cm
**Tree09**	**D30**	−0.030NS	−0.129NS	−0.188NS
	**D60**	0.166NS	0.091NS	−0.168NS
	**D90**	0.187NS	0.253NS	0.031NS
**Tree11**	**D30**	0.611***	0.718***	0.484**
	**D60**	0.181NS	0.467**	0.284NS
	**D90**	0.164NS	0.412*	0.242NS

P values: *P < 0.05; **P < 0.01; ***P < 0.001; NS not significant.

On the other hand, a significant correlation between sonic speed and root diameter was obtained only for shallow soil layers (D30) and at 80 cm distance from the trunk for Tree11 (Table 3), but this correlation had r values that were markedly lower than those for root biomass density.

**Table 3:** Correlation coefficient (r) and *P* values of the correlation analysis (Pearson test) between the sonic speed and root diameter revealed by Root Detector and Fastrak, respectively, in two olive trees at different distance from trunk (0–40, 0–80 and 0–120 cm) and soil depth (D30, D60, D90).

		0–40 cm	0–80 cm	0–120 cm
**Tree09**	**D30**	0.211NS	−0.189NS	−0.378*
	**D60**	−0.003NS	−0.143NS	−0.181NS
	**D90**	−0.057NS	−0.070NS	−0.110NS
**Tree11**	**D30**	−0.195NS	0.376*	0.325NS
	**D60**	−0.191NS	0.302NS	0.051NS
	**D90**	−0.215NS	0.303NS	−0.009NS

P values: *P < 0.05; **P < 0.01; ***P < 0.001; NS not significant.

The strict root biomass and sonic speed relationships that were observed more for Tree11 than for Tree09 and at shallower (D30) but not at deeper (D60 and D90) soil layers are highlighted in the visual comparison of the radial distribution of the total root biomass density shown in Fig. 6 and the sonic speed shown in Fig. 2. Indeed, at the D30 soil depth, the sonic speed was higher for Tree11 than for Tree09, and it decreased with increasing distance from the trunk, as observed for the root biomass density. To better represent this relationship, the radial distribution of the sonic speed and root biomass density values for the olive trees in the 36 sectors of 10° degrees at different distances from the trunk and at D30 and D90 soil depths were normalized, respectively, where the maximum values were assigned a value of unity (Figs. S5 and S6). For Tree11, the radial distribution of the root biomass density coincided with that of sonic speed values but at the D30 soil depth only (Fig. S5), while this overlapping was lost at the D90 soil depth (Fig. S6). Conversely, the radial distribution of the root biomass density of Tree09 did not overlap with the sonic speed values at any distance from trunk or any soil depth (Figs. S5 and S6). Although referred to the investigated trees, it is reasonable to scale the reliability of the Root Detector to a distance of up to 6-fold the DBH (measured at 1 m height in this specific case study) and to a soil depth of 30 cm provided that more than 35% of the total root biomass occurs within this range.

## Discussion

The two studied olive trees exhibited completely different root architecture in terms of the radial and depth distribution of the root biomass; the roots were shallower for Tree11 and deeper for Tree09. These two different root distribution patterns, on the one hand, confirmed the results of Sorgonà *et al*^24^ noting a root biomass distribution for mechanically harvested olive trees (i.e., Tree11) that was more superficial than that of manually harvested trees (i.e., Tree09); on the other hand, these patterns were suitable for testing the accuracy and limits of Root Detector technology.

In this study, a quantitative evaluation, i.e., a correlation analysis between the sonic speed and the root diameter values within each 10° sector at different soil depths and distances from the trunk for each olive tree, was performed. The sonic speed values were higher than those obtained by Rinn (2016) (0–500 m s^-1^) by sonic tomography with Arboradix (Rinntech, Heidelberg/Deutschland). The different plant species, environmental conditions, soil moisture content and soil compaction levels and, above all, the different instruments are probably involved in this difference^48,49^.

The first question addressed by this work was to verify through a statistical approach the capacity of the Root Detector to reveal the presence of coarse tree roots at a certain soil depth and distance from the trunk. For these reasons, the sonic speed values obtained by Root Detector were correlated with the root biomass density measured by Fastrak for each 10° sector at different soil depths and distances from the trunk of each olive tree. These results indicated that the Root Detector revealed the root architecture within 30 cm of soil depth and 120 cm of distance from the trunk, confirming the findings of Rinn^39^ and Buza and Goncz^50^. For example, by qualitative assessment, Rinn^39^ suggested that the higher the sonic speed, the shallower and larger the roots, while Buza and Divos^40^ reported that when a root is nearby (closer than 10 cm), the sonic travel time decreases significantly. Indeed, as observed in this study, the clear detection of the root architecture of Tree11 was possible because it was characterized by a shallower root architecture than Tree09, which conversely deepened its root axes in the soil, as revealed by the Fastrak method (Fig. 6).

The second question addressed by this work was to identify the minimum root diameter revealed by Root Detector and whether root diameter or biomass density was better detected. Both Rinn^39^ and Divos and Buza^40^ suggested that the Root Detector resolution is 3 cm in diameter, but these authors used a qualitative assessment after the excavation. The results from this study suggested that the Root Detector detected the root biomass density more efficiently than the root diameter. In wood, sonic waves travel through the wood grains and vary with grain angle, including grain angles due to knots/laterals and spiral grain. In particular, the value of the longitudinal component of the sonic wave is 20-fold higher than that of the radial wave^51,52^, i.e., the greater the longitudinal component, the higher the speed. Moreover, the speed may increase with increasing tissue density (g dry biomass cm^−3^) and decreasing moisture content (^52^ and references therein). All these characteristics of wood properties make tissue density more important for sonic wave propagation than diameter, as tissue density may remain constant with changing diameters. The lack of correlation with the diameter makes the minimum diameter impossible to identify or obtain without allometric calculations. Furthermore, at greater distances from the trunk, the increased soil volume within which the root axes were located diminished the root biomass density, causing a reduction in the sonic speed. The markedly lower magnitude of the radial component of the sonic wave further explains the limited detection of the deeper roots.

The third question is related to the ability of the Root Detector to determine the root radial distribution of the tree roots. Correlation analysis (Table 2) suggested that the Root Detector permits us to reveal the radial direction of the olive roots as long as they are located within 30 cm of soil depth and 120 cm of distance from the trunk.

In conclusion, these outcomes clearly showed that the Root Detector detected olive roots in the soil environment, but only under certain conditions. First, the shallower (within 30 cm of soil depth) the root axis deployment, the better the resolution of the Root Detector. Moreover, the detection of the roots was independent of the distance from the trunk (40, 80 and 120 cm) as long as the roots were located within 30 cm of soil depth, although the sonic speed diminished with these distances. In addition to the simple presence of roots in the soil, this study also noted that Root Detector technology fails to detect the root size of olive trees in terms of geometric parameters such as root diameter.

Overall, this non-destructive technique could be useful in forestry and ecology for evaluating the safety factor for uprooting the root system of individual trees, as shown by Rinn^39^ and Buza and Divòs^40^, and it could also be applied to urban trees for the safety of citizens and civil buildings. Indeed, despite the aforementioned limitations, the detection of surface horizontal roots is very important for tree stability, as they increase the turning moment for uprooting and root-soil plate rigidity by increasing the radial distance of the root-soil plate hinge^53^. Anchorage strength measurements on 18–40 cm DBH and 12–27 m height conifer trees^54^ and both conifer and dicot temperate trees of on average 46 cm mean DBH and 33 m height^55^ revealed the occurrence of the hinge (or rotation) axis within 1 m from the trunk base despite a root-soil plate with a surface radius of 1.0–1.6 m. Such a hinge distance falls within the range investigated in this study (up to 1.2 m) despite the smaller dimension of the selected olive trees. Therefore, although limited to two sampled trees, the selected distances and the clearly different root architectures investigated may be considered a valid trial for assessing the contribution of surface structural roots to the safety evaluations. Undoubtedly, the expanded use of this technology for various tree species with different root system architecture patterns would benefit from continued efforts at validation.

## Supplementary information

Supplementary information

## Data Availability

The datasets generated during and/or analysed during the current study are available from the corresponding author on reasonable request.
