# Supplementary material for: A sonic root detector for revealing tree coarse root distribution
Source: Sci Rep. 2020 May 15;10:8075. doi: 10.1038/s41598-020-65047-4 (PMC7228922; doi:10.1038/s41598-020-65047-4)
Supplement: Supplementary file 1 — Supplementary information [file 41598_2020_65047_MOESM1_ESM.pdf]

Scientific Reports

A sonic root detector for revealing tree coarse root distribution

Andrea R. Proto, Antonino Di Iorio *, Lorenzo M. Abenavoli, Agostino Sorgonà

*corresponding author; antonino.diiorio@uninsubria.it

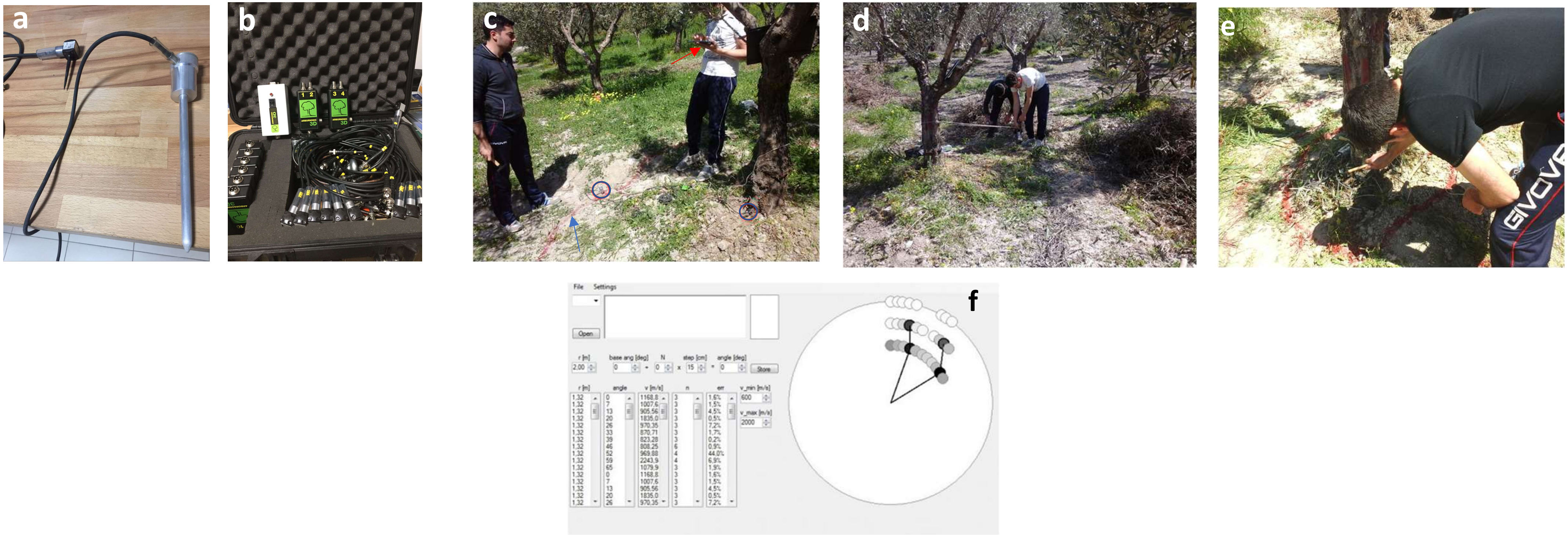

Fig. S1 The root detector components, work procedure and output.

a) The transmitter (SD02, piezo sensor) and the receiver (soil sensor, a high frequency geophone); b) the control unit (ArborSonic 3D); c) the spikes of the transmitter and receiver inserted in the tree collar (the tapping point) and the soil, respectively (blue circles), the distance from trunk marked using lines on the ground (arrow in blue colour) and the tablet with the ArborSonic software package (arrow in red colour); d) the loose rope knotted around the tree. The rope will help you keep constant distance between the tree and soil sensor; e) The hit on the transmitter sensor; f) graphical output

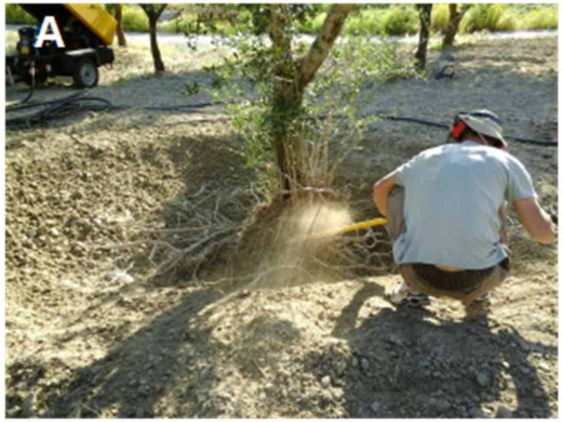

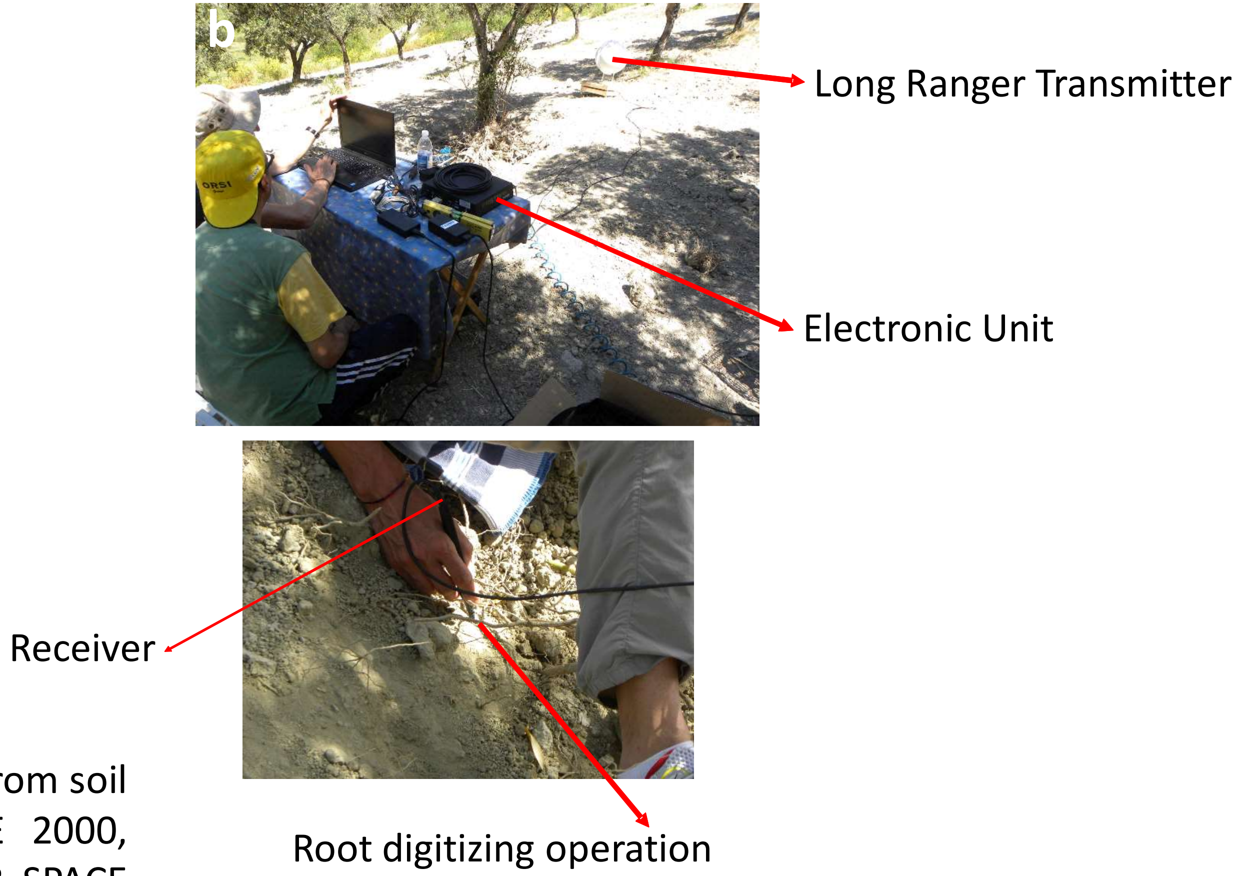

Fig. S2 a) Freeing of olive tree root system from soil using high-pressure air lances (Air-SPADE 2000, Chicopee, MA, USA); b) the 3D digitizer (3 SPACE Fastrak, Polhemus) constituted by the electronic unit, the magnetic transmitter (Long Ranger), and the small hand-held receiver.

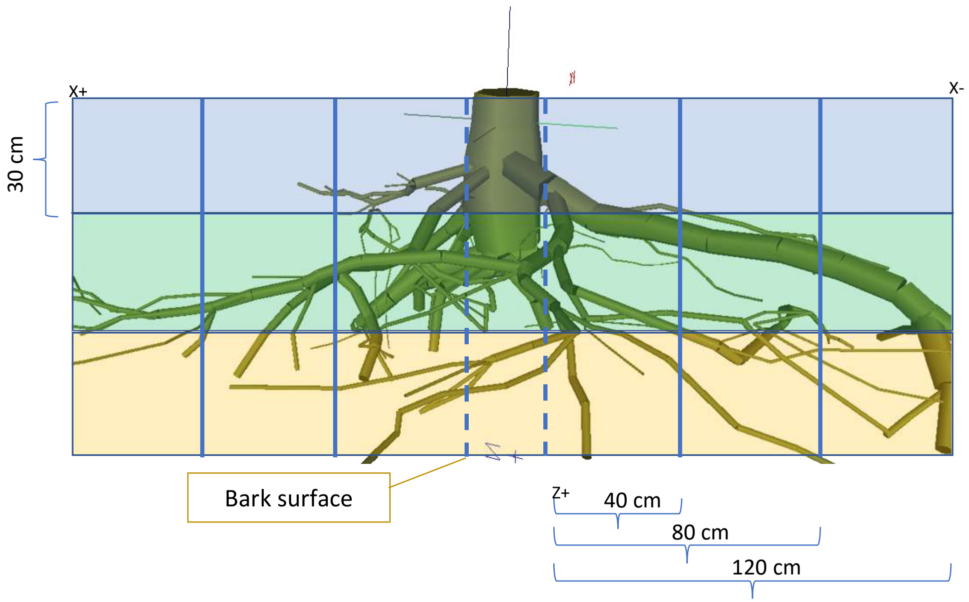

Fig. S3 Sketch of the virtual grid within which all first-to-third-order lateral roots were virtually sliced, and their volume determined. Root system image was obtained from digitizing with AMAPmod software. Lateral view showing the virtual soil boxes 30 cm thick and 40 cm wide stacked to 90 cm depth and 120 cm distance.

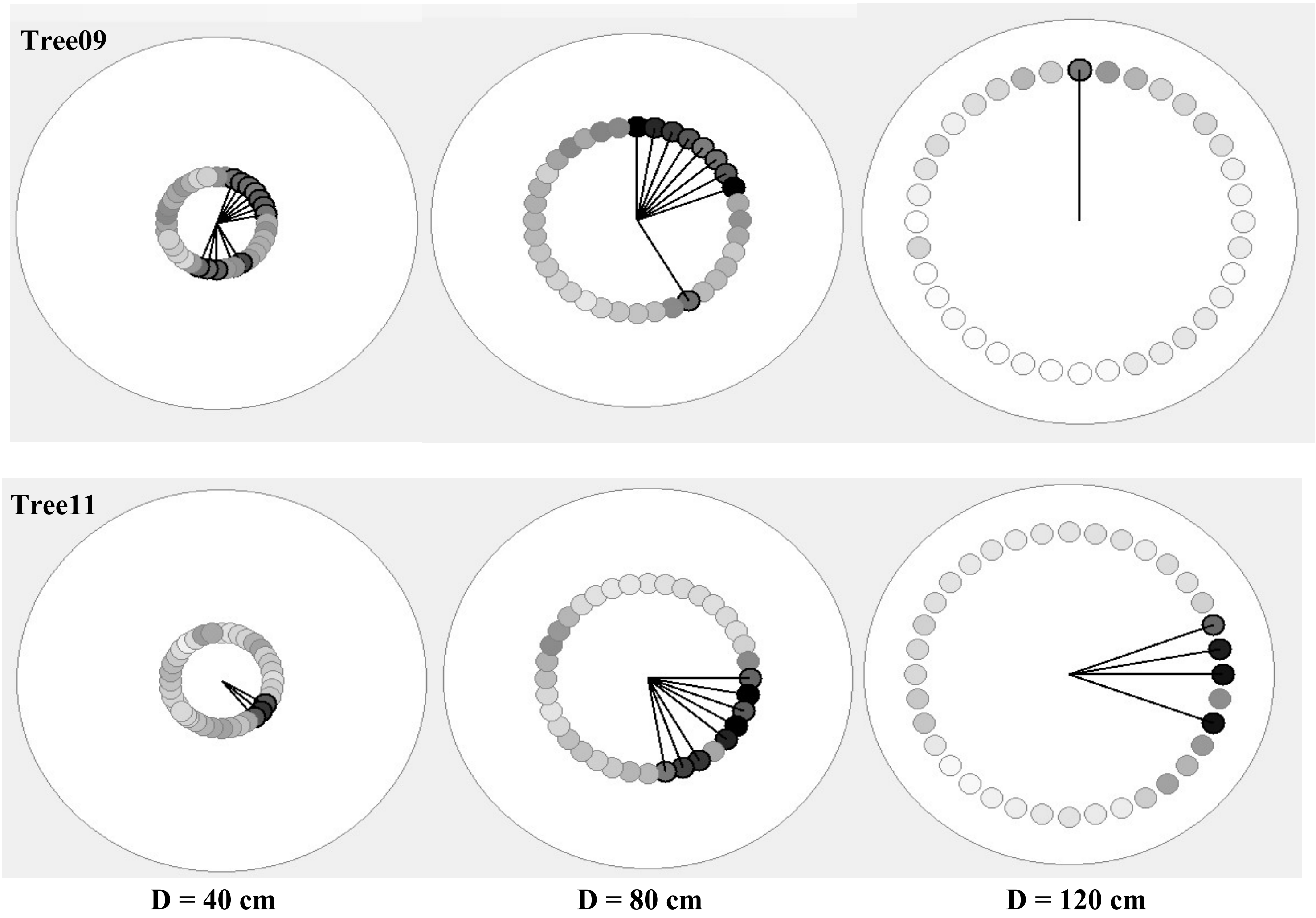

Fig. S4 Graphical visualization of the Root Detector Evaluation Software (Fakopp Enterprise Bt, Hungary) for *Olea europea* trees (Tree09 and Tree11) at different distance from the bark surface (40, 80 and 120 cm). Grey levels refer to the sonic speed, i.e. the darker the level the higher the speed. Lines highlight directions from the hypothetical center of the trunk to the points whose speed values are higher than the mean value of the measured range (min+max)/2

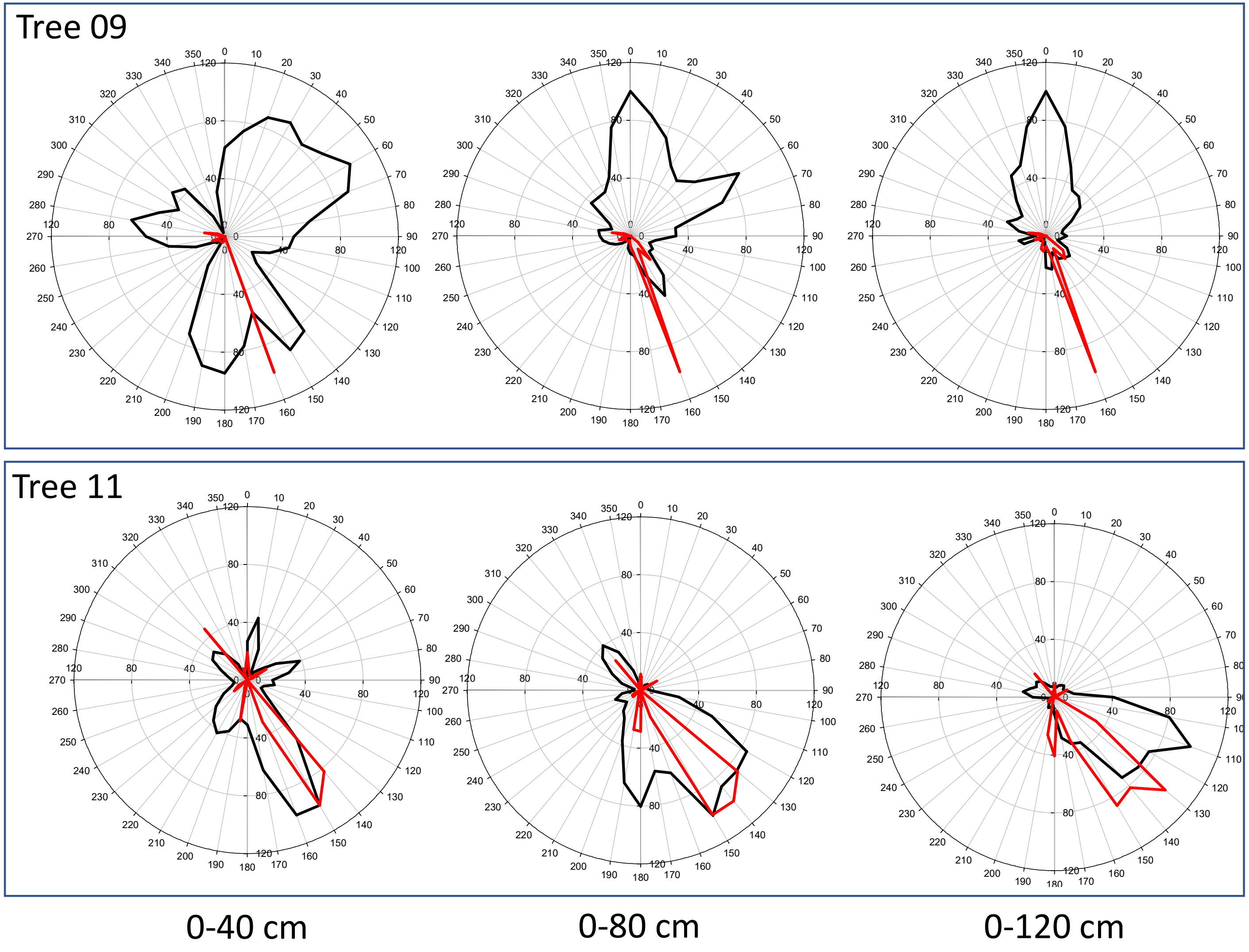

Fig. S5 Sonic speed (m s^{-1}) (black line) measured by Root Detector and root biomass density (kg m^{-3}) (red line) measured by Fastrak to 30 cm soil depth at different distances from the trunk (40, 80 and 120 cm) and in 36 sectors 10 degree wide for the two studied *Olea europea* trees. The data are normalized to the respective maximum value.

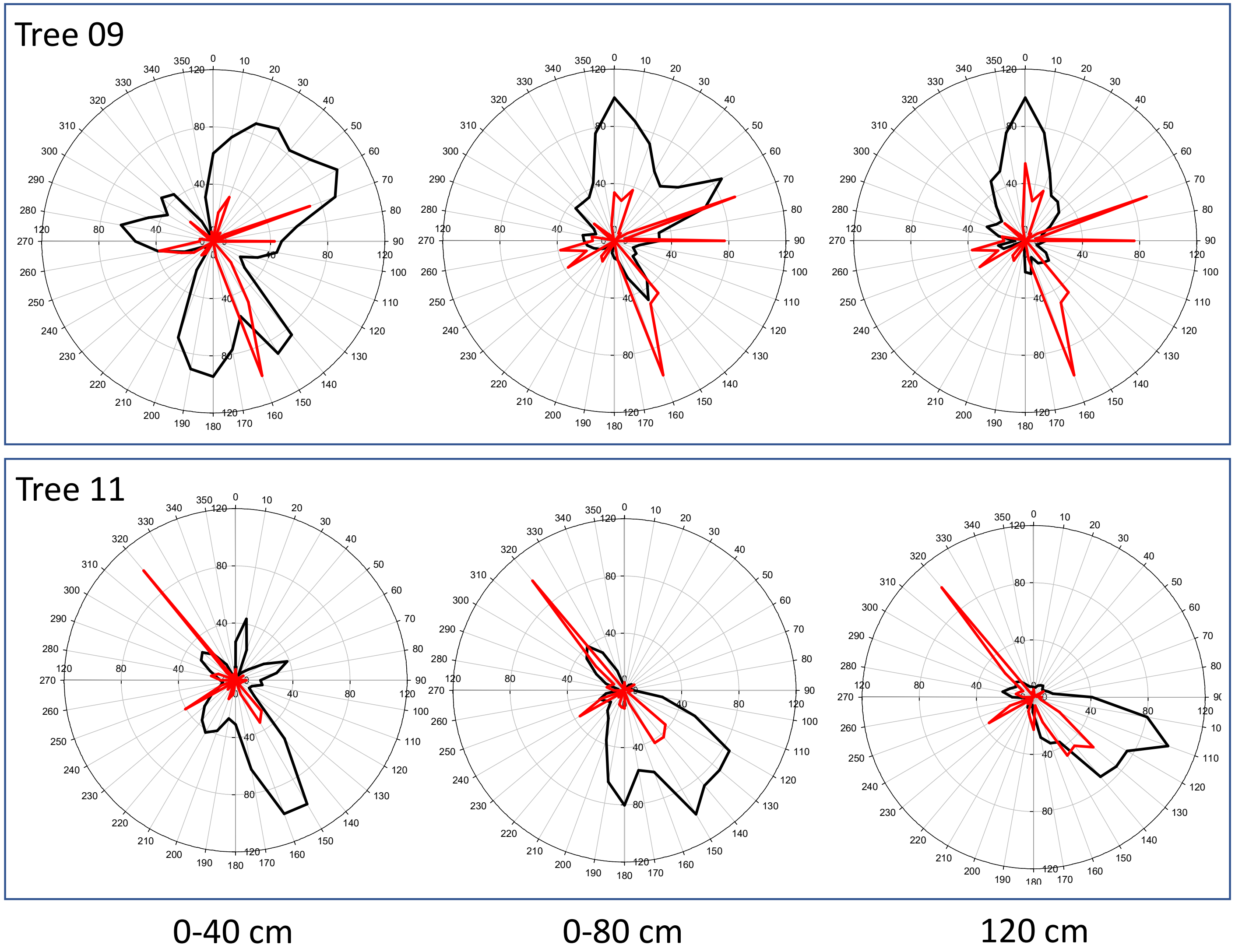

Fig. S6 Sonic speed (m s^{-1}) (black line) measured by Root Detector and root biomass density (kg m^{-3}) (red line) measured by Fastrak to 90 cm soil depth at different distances from the trunk (40, 80 and 120 cm) and in 36 sectors 10 degree wide for the two studied *Olea europea* trees. The data are normalized to the respective maximum value.
